# Impact of one-rod levonorgestrel implant on the blood chemistry profile

**DOI:** 10.1038/s41598-021-99801-z

**Published:** 2021-10-11

**Authors:** Eka R. Gunardi, Raymond Surya, Inayah Syafitri, Yogi Pasidri

**Affiliations:** Department of Obstetrics and Gynecology, Faculty of Medicine, University of Indonesia—Cipto Mangunkusumo General Hospital, Jakarta, Indonesia

**Keywords:** Clinical trials, Randomized controlled trials

## Abstract

The aim of this study was to investigate the effect of a one-rod levonorgestrel implant on the blood chemistry profile, including random blood glucose (RBG), haemoglobin (Hb), alanine transferase (ALT), aspartate transferase (AST), and the lipid profile, including total cholesterol, high-density lipoprotein (HDL), low-density lipoprotein (LDL), and triglycerides. This prospective cohort study was conducted at Raden Saleh Clinic, Jakarta, from 2010 to 2012. The implants were inserted subdermally in 30 patients. The subjects were evaluated every 6 month up to 2 years. Bivariate analysis using t-test or Wilcoxon signed rank test was performed for all variables. p < 0.05 was considered a significant value. The Hb, RBG, AST, and lipid profile levels were significantly different before and 6 months after one-rod implant insertion (p < 0.05). However, for 24 months, all of the parameters were still within normal limits and did not differ clinically. One-rod levonorgestrel implant insertion has a minimal effect on all blood chemistry profiles.

## Introduction

A long-term contraceptive device that has been widely used is the hormonal implant. The hormonal implant is a contraceptive device that is inserted subdermally in the medial region of the upper arm and contains steroidal hormones. There are several kinds of progestogens that may be used in hormonal implants, one of which is levonorgestrel^1^.

Levonorgestrel, as a second-generation synthetic progestin and gonane structure derivative, has been widely used as an active compound in hormonal implants. The hormones are slowly released into circulation at the same concentration over a 5-year period. Levonorgestrel levels will rise quickly in the first month after insertion and slowly decrease until the expiration. The hormonal level is also influenced by the subject’s body weight. The side effects of synthetic progestin are oedema, abdominal bloating, anxiety, irritability, depression, and myalgia^2,3^. Monoplant is a hormonal implant that contains 160 mg of levonorgestrel and was developed in Indonesia. Monoplant was estimated to have a contraceptive effect for up to 3 years^4^.

Hormonal contraceptives, especially combined hormonal contraceptives, are known to affect metabolism and induce changes in lipid, lipoprotein, carbohydrate, and haemostatic factor levels^5^. The metabolic impact of progestin contraceptives has not been investigated thoroughly. Subdermal implant contraceptives are a type of contraceptive that releases low-dose progestin continuously, and implant contraceptives are a type of contraceptive that is continuously developing. Recently, a one-rod levonorgestrel (LNG) implant contraceptive (Monoplant) has been developed^6^. The aim of this study was to investigate the effect of a one-rod levonorgestrel implant on the blood chemistry profile, which includes haemoglobin (Hb), random blood glucose (RBG), aspartate aminotransferase (AST), alanine aminotransferase (ALT), and the lipid profile including total cholesterol, high-density lipoprotein (HDL), low-density lipoprotein (LDL), and triglycerides.

## Methods

This prospective cohort study was conducted at Raden Saleh Clinic, Jakarta, which is a satellite of Dr. Cipto Mangunkusumo Hospital, from 2010 to 2012. This analytical study with a prospective cohort design aims to assess the effect of one-rod levonorgestrel implants on blood chemistry profiles, including Hb, RBG, AST, ALT, and the lipid profile (total cholesterol, HDL, LDL, and triglycerides). This study was a phase two clinical study, in which one-rod levonorgestrel implants were assessed for the first time in patients. This study was evaluated every 6 month up to 24 months.

The minimum sample size was calculated with a chosen type I error rate of 0.05 and power of 80%, resulting in a minimum of 30 individuals in each group. The subjects in this study were women of reproductive age (20–40 years old) who planned to delay or space their pregnancies. The hormonal status, especially related to the ovulation process, was examined for each participant. The blood chemistry profile in this study was assessed in Dr. Cipto Mangunkusumo Hospital. The technique was guaranteed as an international standard procedure. The blood chemistry profile was assessed before implant insertion. Every subject was then followed, and the blood chemistry profile was examined at the 6th, 12th, 18th, and 24th months.

The inclusion criteria were women aged 20–40 years who engaged in regular sexual activities, did not use any hormonal contraceptives for the last 6 months, provided informed consent for the study, and were able to participate in the routine examination according to the schedule. The exclusion criteria were women who had an abortion in the last two weeks, had not menstruated after delivery, had not menstruated after stopping oral or injection contraceptives, or participated in other studies during the last 6 months. Other exclusion criteria were the presence of any disease that affects the blood chemistry profile (pelvic inflammatory disease since the last delivery, history of ectopic pregnancy, malignancy, abnormal bleeding, abnormal menstrual cycle, amenorrhea, breast abnormalities, thromboembolism, mental illness including depression, headache, or diabetes mellitus). The study was conducted at Raden Saleh Clinic, Jakarta, from 2010 to 2012.

The implants were inserted subdermally during the first to seventh day of menstruation. The subjects were followed for 6 months with monthly observation. During those periods, Hb, RBG, AST, ALT, and lipid profiles were tested.

Every subject had received an explanation about the study and agreed to be participants. Every subject that was included in this study was given and signed the informed consent form. This study was approved by the ethics committee of the Faculty of Medicine, University of Indonesia (number 176/PT02.FK/ETIK/2009). All experiments were performed in accordance with relevant guidelines and regulations.

### Statistical analysis

Descriptive distributions are shown as frequencies and percentages. Bivariate analysis using t-test or Wilcoxon signed rank test was performed for all variables. A p value < 0.05 was considered significantly.

## Results

A total of 30 subjects who were included in this second phase of the clinical study did not get pregnant until the end of the study. There were no drop out subjects during the follow up. The baseline characteristics of the subjects are shown in Table 1.

**Table 1:** Baseline characteristics of subjects.

Variables	N (%); Mean ± SD; Median (Min–Max)
Age (year)	31.6 ± 5.5
**Education**	
Elementary	6 (20%)
Junior high school	6 (20%)
Senior high school	16 (53.3%)
College graduate	2 (6.7%)
Parity	2 (1–5)
**History of contraception**	
Hormonal injection	18 (60%)
Hormonal pill	5 (16.7%)
Hormonal implant	3 (10%)
Intrauterine device	1 (3.3%)
Others	1 (3.3%)
No history	2 (6.7%)
Body mass index (kg/m^2^)	22.9 ± 4.4

The mean Hb, RBG, AST, ALT, and lipid profile (total cholesterol, HDL, LDL and triglyceride) values before and six months after implant insertion were still in the normal range (Table 2). The levels of Hb, RBG, AST, total cholesterol, LDL, and HDL cholesterol were significantly different before and 6 months after one-rod implant insertion (p < 0.05).

**Table 2:** Blood chemistry profile before and 6 months after one-rod implant insertion.

Blood chemistry	Before (n = 30)	After (n = 30)	Normal range	p
Hb (g/dL)	13.5 ± 1.2	12.8 ± 0.8	12–14	**0.005**^a^
RBG (mg/mL)	78.6 (55–142)	78.5 ± 17	< 160	0.281^b^
AST (mU/mL)	22.4 ± 6.5	30.5 (10–94)	< 31	**0.006**^b^
ALT (mU/mL)	16.5 (9–53)	20.5 (7–93)	< 31	0.128^b^
Total cholesterol (mg/mL)	176.6 ± 22.4	180 ± 23.2	140–250	0.460^a^
LDL (mg/mL)	105.9 ± 20.6	95.8 ± 21	< 155	**0.023**^a^
HDL (mg/mL)	47.5 (33–74)	61.4 ± 11.4	> 35	** < 0.001**^b^
Triglycerides (mg/mL)	110.5 (62–314)	90.5 (60–326)	< 200	0.434^b^

Bold values indicate when the levels of Hb, RBG, AST, total cholesterol, LDL, and HDL cholesterol were slightly increase or decrease but still in a normal range.

^a^Paired T-test.

^b^Wilcoxon test.

The mean Hb level during the study was normal (12–14 g/dL). The mean RBG level remained at a low level, which was below < 160 mg/mL. The liver function parameters including AST and ALT (< 31 mU/mL), during the observation period were normal. Similar results were observed for the lipid profile, including total cholesterol, HDL, LDL, and triglycerides (Table 3).

**Table 3:** The blood chemistry profile during 24 months of follow-up.

Blood chemistry	Before (n = 30)	6th month (n = 30)	12th month (n = 30)	18th month (n = 30)	24th month (n = 30)	Normal range
Hb (g/dL)	13.5 ± 1.2	12.8 ± 0.8	13.3 ± 0.6	12.6 (10.2–38.2)	13 ± 0.8	12–14
RBG (mg/dL)	78.6 (55–142)	78.5 ± 17	74.8 ± 11.4	83.7 ± 15.5	96.8 ± 16.2	< 160
AST (mU/mL)	16.5 (9–53)	20.5 (7–93)	23.8 ± 6.6	19 (10–98)	18 (13–39)	< 31
ALT (mU/mL)	22.4 ± 6.5	30.5 (10–94)	20 (8 – 48)	15 (8–43)	16.1 ± 5.3	< 31
Cholesterol (mg/mL)	176.6 ± 22.4	180 ± 23.2	169.6 ± 23.9	160 (12–206)	178.5 (133–340)	< 200
HDL (mg/mL)	105.9 ± 20.6	95.8 ± 21	51.3 ± 9.4	41 (34–96)	42.7 ± 8.8	> 35
LDL (mg/mL)	47.5 (33–74)	61.4 ± 11.4	95.2 ± 22.5	94 (39–140)	114.5 (77–245)	< 155
Triglycerides (mg/mL)	110.5 (62–314)	90.5 (60–326)	97.5 (11–295)	102 (60–315)	113.5 (67–307)	140–250

## Discussion

This study aimed to assess the effect of one-rod LNG implants on blood chemical indices, such as Hb, RBG, AST, ALT, and the lipid profile. Based on the results, the total cholesterol level was significantly different before and 6 months after one-rod implant insertion. Observations over two years revealed that there was an increasing trend in total cholesterol. However, it was not clinically significant. This result was similar to the study by Affandi, which investigated a six-rod 36 mg LNG implant (Norplant) in a cross-sectional study^3^. However, this result was different from most studies that investigated the effect of levonorgestrel on the lipid profile. The total cholesterol level usually decreased significantly during levonorgestrel implant use. Apart from that, progestin-only contraceptives method was not robustly associated with metabolic perturbations. Only contraceptive containing estrogen is responsible for broad metabolic effects such as a higher risk of venous thrombosis, thrombotic stroke, and myocardial infarction^7–10^.

The HDL cholesterol level in this study decreased significantly. Studies investigating the effect of levonorgestrel on HDL cholesterol levels have shown variable results. Several studies have reported a significant decrease in HDL cholesterol levels. Three studies reported no change in HDL cholesterol levels. Other studies reported no change during the first 9 months but a slight significant increase at the 12th month. In other studies, they reported a significant increase 6 months after insertion compared with the level in the control IUD group; however, there was a decrease in the 12th to 24th months, although it was not significant. Finally, a study with a follow-up period of 5 years in Singapore found that the HDL cholesterol level changed variably over time. A significant increase was observed 12 months after implant insertion and 6 months after extraction, while there was a significant decrease observed in the third and fourth years and a similar level at the beginning of the second and fifth years^11^. Dilbaz B et al. stated that etonogestrel implants, a derivate of progestogen, were associated with a decrease in total cholesterol and HDL cholesterol^12^. However, the decrease in HDL cholesterol was unlikely to be related to increased cardiovascular function because in this study, HDL at 2 years was still in the normal reference range.

All the blood chemistry parameters in this study were in the normal range two years after one-rod implant use. This result was not different from similar studies investigating the effect of LNG on the blood chemistry profile, which is usually not constant. Taheri et al. found that cholesterol and triglyceride levels were unchanged, while AST and ALT levels increased slightly^13^.

Dorflinger^5^ investigated the metabolic effects of several implant contraceptives, including LNG, etonorgestrel, nomegestrol acetate, and Nestorone, on liver function, kidney function, glucose, insulin, haemostasis, and blood pressure. The overall metabolic effects were reported to be minimal. Every parameter in the study was still in the normal range for the study population and changes were not clinically significant. In addition, Bender et al. showed changes in fasting glucose and insulin sensitivity among obese individuals using progestin-only contraceptive methods. Therefore, the benefits should be weighed against the risk for this change in metabolic markers^14^.

Based on the results above, we considered that this one-rod LNG implant (Monoplant) was safe for use because it was not associated with clinical differences during a two-year follow-up. The strength of this study is that it was the first to assess the effect of a one-rod LNG implant (Monoplant) on the blood chemical profile with a prospective cohort approach and similar subjects. However, the factors that influence metabolic markers such as diet, lifestyle, and medication were not assessed, which is a limitation of this study. Furthermore, this study should measure fasting blood glucose or HbA1c because RBG is highly influenced by several factors. This study was part of a previous phase II clinical trial and can be used as a basis for a phase III clinical trial in the future.

## Conclusion

The insertion of a one-rod levonorgestrel implant can provide an optimal effect in preventing pregnancy. In addition, this one-rod levonorgestrel implant showed minimal effect on all blood chemistry profiles after 2 years of follow-up.
